# Qualitative Evaluation of Educational Content on Lateral Spine Surgery YouTube™ Demonstrations

**DOI:** 10.7759/cureus.29591

**Published:** 2022-09-26

**Authors:** Periklis Godolias, Kaarina Charlot, Angela Tran, Jonathan Plümer, Charlotte Cibura, Zeyad Daher, Marcel Dudda, Thomas A Schildhauer, Jens Chapman, Rod J Oskouian

**Affiliations:** 1 Orthopedics and Trauma Surgery, St. Josef Hospital Essen-Werden, Essen, DEU; 2 Spine Surgery, Seattle Science Foundation, Seattle, USA; 3 Orthopedics and Trauma Surgery, BG University Hospital Bergmannsheil, Ruhr University Bochum, Bochum, DEU; 4 Trauma, Hand, and Reconstructive Surgery, University Hospital Essen, Essen, DEU; 5 Orthopedics and Trauma Surgery, BG University Hospital Bergmannsheil, Bochum, DEU; 6 Neurosurgery, Swedish Neuroscience Institute, Seattle, USA

**Keywords:** olif, xlif, llif, lateral, social media, youtube™

## Abstract

Objective: This study assessed the quality of educational content for lateral spine fusion procedures on YouTube™.

Methods: YouTube™ was searched using the following keywords and phrases:* *“Lateral lumbar interbody fusion,” “lateral lumbar spine surgery,” “Oblique lateral interbody fusion (OLIF),” “Extreme lateral interbody fusion (XLIF),” and “Lateral lumbar interbody fusion (LLIF).” An expert panel of three senior-level spine surgeons [rater one to three (R1-R3)] rated videos on 13 qualitative evaluation parameters via a modified Delphi approach.

Results: Thirty-eight videos were included for evaluation. Interrater reliability analysis indicated a moderate agreement between R1 and R2 (κ=0.50; standard error, SE = 0.05), R1 and R3 (κ = 0.60, SE = 0.04), and a substantial agreement between R2 and R3 (κ = 0.65, SE = 0.04). Unanimously positive assessments of the quality of the intraoperative presentation varied between 42% and 63% of the rated videos. However, perioperative quality features were unanimously rated positively less than 21% of the videos.

Conclusion: With regard to the surgical approach and execution of lateral lumbar fusions, YouTube™ videos can be seen as a valuable addition to academic education. The main problem, however, is the lack of control mechanisms that check the quality of the content offered before it is consumed by patients, students, and doctors in training.

## Introduction

Developed by Briggs and Milligan in 1944, lumbar interbody fusion surgery is traditionally performed via a posterior approach [1]. However, interest in antero-lateral interbody fusion (ALIF) and lateral lumbar interbody fusion (LLIF) approaches for lumbar fusion procedures has emerged due to purported benefits [2-4]. First introduced by Ozgur et al. in 2006, LLIF procedures have become common with subsequent refinement leading to even less invasive approaches such as far lateral transpsoas and prepsoas [4]. Lateral transpsoas and prepsoas approaches along with technological improvements have broadened the indications and rates of LLIF procedures with purported benefits of decreased blood loss, preservation of posterior ligamento-fascial structures, easier access to the disc space, and possibility to place larger interbody cages capable of providing a substantial endplate coverage with the capability to indirectly decompress neural elements and provide a larger fusion bed [5]. Emerging lateral techniques are more challenging than traditional approaches and complications have been observed. LLIF procedures are associated with injury to the vessels around or proximal to the lumbar spine, ureter injuries as well as bowel injuries along with the potential for increased procedural costs, relative to more traditional techniques [6]. With the continued expansion of digital media, medical professionals have increasingly turned to online resources for ad hoc immediate educational resources [7]. Search engines like Google, Bing, and Yahoo provide a vast amount of information on a variety of different surgical procedures [7-8]. However, concerns have been raised regarding the quality of materials regarding LLIF procedures on some online sites [9]. Over time, YouTube™ has become the most utilized online source for educational content. A 2016 survey study among fourth-year medical students, residents and faculty surgeons in General Surgery, identified YouTube™ as their main video-based platform in preparation for procedures [9]. Easily accessible teaching videos for surgical procedures can provide multimedia exposure through the incorporation of pictures and text materials for more engaging and comprehensive content delivery [10-11]. More current publications suggest that video instruction can be a viable tool for the development of both clinical and surgical skills, and provide structured teaching modules for medical professionals [12-14]. While some studies have assessed the general informative nature of YouTube™’s videos and the potential benefit they offer for surgical preparation, a more dedicated and structured evaluation of available educational videos on LLIF-type surgery has to date not been performed. In this study, our primary aim was to identify the extent of educational content for LLIF procedures on YouTube™, as well as analyze the quality of these instructional videos utilizing a modified Delphi approach.

## Materials and methods

We used the Google Chrome web browser. The YouTube™ database was searched using the following terms deemed most relevant to the topic of study: “Lateral lumbar interbody fusion,” “lateral lumbar spine surgery,” “Oblique lateral interbody fusion (OLIF),” “Extreme lateral interbody fusion (XLIF),” and “LLIF.” Results were listed in order of YouTube™’s algorithm, and the total number of results was recorded for each term. The first 100 results for each of the five terms were included in the initial review. Initial review inclusion criteria included videos narrated in English, videos made for surgeon education, and videos created within or associated with a healthcare institution. Videos were excluded if they were presented in lecture format, largely consisted of audiovisual animations, did not include audio, or were narrated in a language other than English. Quantitative data were collected for each video, including the total number of views, video length, as well as number of likes and dislikes. The total amount of results and videos included for each search phrase are shown in Table 1.

**Table 1:** Summary of search results for each search phrase. OLIF, oblique lateral interbody fusion; XLIF, extreme lateral interbody fusion; LLIF, lateral lumbar interbody fusion

Search phrase	Total results	Included
Lateral lumbar interbody fusion	24800	12
Lateral lumbar spine surgery	10100	7
OLIF	27400	7
XLIF	63500	11
LLIF	11000	1

Qualitative evaluation parameters were determined by three senior-level spine surgeons (R1; R2; R3) via a modified Delphi approach, each of which was deemed integral to determining the educational value of each video. Each surgeon had more than 20 years of operative experience in spine surgery and had been performing lateral approaches for at least 10 years. The parameters included essential pre-operative, intra-operative, and post-operative surgical factors deemed relevant for safe and effective lateral lumbar spine surgery. The set selection criteria did not limit any presented techniques, instead, emphasis was placed on the efficacy of communicating integral surgical indications, procedures, and any other relevant details. Thirteen total evaluation characteristics were outlined, falling into three distinct categories -- intra-operative presentation quality, perioperative management, and audiovisual presentation clarity. Our evaluation parameters are further outlined in Table 2.

**Table 2:** Scoring criteria for each video.

Intraoperative quality criteria		Yes	No
1	Correct patient positioning shown	I	0
2	Appropriate level and skin incision shown	I	0
3	Approach shown with attention to structures at risk	I	0
4	Visualization of the surgical approach	I	0
5	Fluoroscopic guidance and visualization of the approach	I	0
Pre/postoperative quality criteria			
6	Indications discussed	I	0
7	Patient selection discussed	I	0
8	Alternative surgical/non-surgical methods discussed	I	0
9	Postoperative management discussed	I	0
10	Complications discussed	I	0
Audiovisual metrics			
11	Language is intelligible	I	0
12	Adequate video resolution	I	0
13	Appropriate video angle	I	0

A binary scale was utilized for grading of each video: the video would receive a score of “I” if it satisfactorily addressed a particular parameter, or a score of “0” if it failed to sufficiently address the parameter. Audiovisual quality was graded in a similar manner, with each video designated an “I” if the audiovisual quality was sufficient, or “0” if the quality was too poor to provide educational value. Descriptive analyses were performed using Stata software (StataCorp. 2005. Stata Statistical Software: Release 9.StataCorp LP, College Station, TX). Interrater reliability was assessed using Cohen's kappa coefficient (κ) to determine the degree of consistency in scoring among the three raters.

## Results

**Figure 1:**
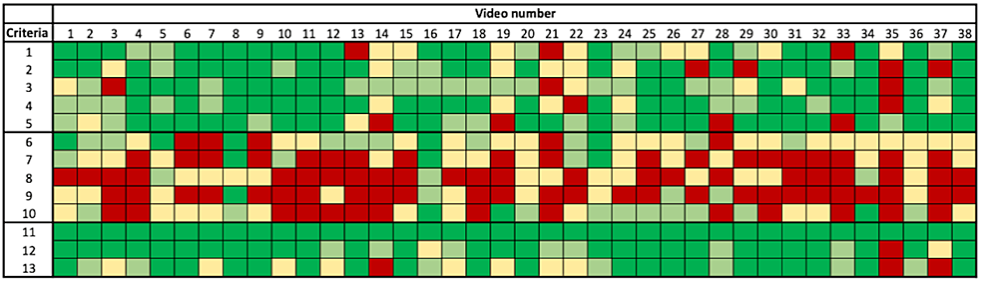
Visualization of consensus of the expert panel. A unanimous agreement was marked in dark green, a majority agreement in light green, a minority agreement in yellow, and a unanimous disagreement in red color.

The number of search results for the various keywords is summarized in Table 1. A total of 136,800 unspecific search results for the five search phrases (lateral lumbar interbody fusion; lateral lumbar spine surgery; OLIF; XLIF; LLIF) were found on the YouTube™ database. The average number of views of the videos was 37.000 (± 23.000). The average number of "likes" and "dislikes" was 1.350 likes (± 368 likes) and 49 dislikes (± 37 dislikes), respectively. The average length of the videos was 12 min (± 9 min). The calculation of Cohen’s kappa coefficients showed a moderate agreement between R1 and R2, and R1 and R3, while a substantial agreement between R2 and R3 was achieved [R1:R2, κ =0.50 (SE 0.05); R1:R3, κ =0.60 (SE 0.04); R2:R3, κ =0.65 (SE 0.04)]. The unanimous consensus of the expert panel on the fulfilment of the quality criteria related to the procedure (Criteria 1-5, Table 2) varied between 42% of the videos regarding the explanation of at-risk anatomical structures during the approach (Criteria 3), and 63% of the videos regarding the fluoroscopic guidance and visualization of the approach (Criteria 5). It was also notable that all three experts agreed that in 61% of the videos alternative surgical or nonsurgical conservative therapy options (Criteria 8) were not mentioned. In a further 31% of the videos, only one of the three experts had the opinion that alternative therapy options had been adequately mentioned. Cumulatively, an overwhelming majority of the videos (92%) showed insufficient to no explanation of treatment alternatives. The expert panel found an even greater consensus with regard to instructions on postoperative management (Criteria 8). All experts unanimously agreed that 68% of the videos did not reference the postoperative management of the patient. Criteria 6-10, which reflect the perioperative management of the patient, were least represented in the videos analyzed. Figure 1 shows a color-coded scheme to illustrate the consensus for all 38 videos included. 

## Discussion

Since YouTube™ first became available online in 2005, the platform has grown steadily and exponentially until it reached two billion reported active monthly users in 2021. However, YouTube™ is no longer just a platform that is used by lay persons for entertainment purposes but has gained everyday utility as an informal audiovisual training platform used by medical students and surgeons of various experience levels as readily available information resources [7, 10, 12]. However, the platform lacks any form of higher-level authority or peer review for filtering inappropriate and incorrect content, which is why a qualitative analysis of the presented content is of great importance [12]. The analysis of the 38 included videos on the search terms "lateral lumbar interbody fusion; lateral lumbar spine surgery; OLIF; XLIF; LLIF," carried out by three senior level spine surgeons, showed an overall satisfactory quality of the surgical measures presented with regard to the surgical procedure itself. With regard to the more complete perioperative management of the patient, including a review of alternative operative and non-surgical treatment methods, the expert group agreed, with moderate to significant consensus that these management aspects were inadequately, or not at all dealt with in the videos analyzed. Although alternative treatment modalities may not be the focus of some instructional videos, the authors felt that preoperative decision-making is an important aspect of lateral lumbar fusion surgery due to its considerable nuances brought on by anatomic and patient variables. Rössler et al. examined the medical information and the qualitative performance of lumbar punctures on YouTube™ and came to the conclusion that only 14% of the rated videos corresponded to all five quality criteria set by the authors in the performance of a lumbar puncture. Thirteen percent of the lumbar puncture videos rated were rated as potentially hazardous to a patient by the authors with regard to the safe execution of this procedure [13]. In contrast, our study showed no concerning deviations from accepted safe practices for lateral lumbar fusions. The authors of a formal literature review from 2019 examined the general quality of educational content of surgical YouTube™ videos. They came to conclusions similar to our results, in that lack of control mechanisms/peer review is a noteworthy deficiency. This is especially applicable to medical professionals in training, like medical students and surgical trainees as these target groups may not be able to differentiate between the appropriate and inadequate quality of teaching content. However, the Farag study did not include any studies in the field of spine surgery [12]. An additional study that examined the informational content of YouTube™ videos on discectomies from a patient's perspective came to a similar conclusion [11]. In general, the existing literature on the subject of surgical YouTube™ training content lacks content and quality control as anyone who has access to this platform can upload any kind of content without being subject to peer review -- both a blessing and a curse. As our focused study on a more recently introduced highly specific and potentially demanding lateral approach spine surgery suggests relatively simple measures might improve viewer’s content differentiation. For instance, following a simple ‘structured’ widely accepted academic presentation format with emphasis on differential diagnosis, alternate treatment, and postoperative management might elevate content completeness. Ultimately, a peer review process for certified videos similar to peer-reviewed PubMed/Medline listed literature combined with commentaries might provide welcome reassurance and also more differentiated content delivery. In its current format, the sole selecting criterium is the resonance of the marketplace -- meaning numbers of viewers, and the placement derived by the search engine algorithm ultimately decide on acceptance. One of the limitations of this study is that the sheer number of YouTube™ videos on the subject made it impossible to examine all available videos. Another limitation is that only experts in the field of lateral spine surgery have rated the videos. It would also have been interesting to evaluate the videos from the perspective of the target group (medical students, spine fellows, etc.), possibly in direct comparison to the scores awarded by the experts.

## Conclusions

Surgical YouTube™ educational training videos can serve as a valuable addition to academic education as seen in our study on the surgical technique of lateral lumbar fusions. Most of the videos were rated as incomplete in terms of content by our experienced reviewers, as many perioperative topics were not adequately addressed. Overall, with an increasing amount of unfiltered content, a restructuring of the platform might help elevate content so that more formal medical content directed at patients, students, and medical specialists is delivered by more dedicated and authorized channels.
